# Cell-type specific repertoire of responses to natural scenes in primate retinal ganglion cells

**DOI:** 10.3389/fncel.2025.1600167

**Published:** 2025-08-12

**Authors:** Alexandra Kling, Nora Brackbill, Colleen Rhoades, Alex Gogliettino, Alexander Sher, Alan Litke, E. J. Chichilnisky

**Affiliations:** ^1^Department of Neurosurgery, Stanford University, Stanford, CA, United States; ^2^Physics Department, University of California, Santa Cruz, Santa Cruz, CA, United States

**Keywords:** retinal ganglion cells, primate retina, natural image processing, retinal output diversity, multi-electrode array recordings

## Abstract

At least 20 distinct retinal ganglion cell (RGC) types have been identified morphologically in the primate retina, but our understanding of the distinctive visual messages they send to various targets in the brain remains limited, particularly for naturalistic stimuli. Here, we use large-scale multi-electrode recordings to examine how multiple functionally distinct RGC types in the macaque retina respond to flashed natural images. Responses to white noise visual stimulation were used to functionally identify 936 RGCs of 12 types in three recordings. Each cell type was confirmed by the mosaic organization of receptive fields, and seven cell types were cross-identified between recordings. Responses to thousands of natural images were used to examine the average firing rate kinetics in each RGC type as well as the repertoire of distinct firing patterns that each type produced. The average response across images was highly stereotyped for cells of each type and distinct for cells of different types. The responses to natural images more clearly distinguished certain cell types than did the response to white noise stimulation. Moreover, the full repertoires of firing patterns produced by different cell types, assessed by their latency and duration, were largely distinct in most cases and in some cases non-overlapping. Together these data provide an overview of the diversity of RGC signals transmitted from the primate retina to the brain in natural viewing conditions.

## Introduction

In primates, at least 20 types of retinal ganglion cells (RGCs) convey visual information to diverse brain regions, including the lateral geniculate nucleus, superior colliculus, and pretectum, where it is further processed to support perception and visually guided behavior (Martersteck et al., 2017; Huberman et al., 2008). Much progress has been made in characterizing the visual signaling properties of the numerically dominant primate RGCs (ON and OFF parasol and midget cells) using artificial stimuli, such as white noise, contrast steps, and frequency chirps (Chichilnisky, 2001; Chichilnisky and Kalmar, 2002; Turner and Rieke, 2016; Karamanlis et al., 2023). More recently, the responses of these major cell types to naturalistic images, which contain richer spatial and temporal structure that engages retinal processing in complex ways, have also been examined (Freedland and Rieke, 2022; Brackbill et al., 2020; Karamanlis et al., 2023; Turner and Rieke, 2016), revealing substantial deviations from traditional models of RGC response. However, few studies have examined responses to naturalistic stimuli in the ∼16 or so lower-density RGC types (Karamanlis et al., 2025; Dacey, 2004), which constitute about a third of the fibers in the optic nerve and have different retinal connectivity and patterns of projection in the brain than the four numerically dominant types. Thus, our understanding of visual signaling in natural conditions by the diverse visual pathways emanating from the primate retina remains limited.

As a first step, in this study we characterize the average response and response repertoire of diverse primate RGC types to a large set of naturalistic stimuli. By combining large-scale multi-electrode array recordings with quantitative analysis of response dynamics, we show that the temporal neural code is highly varied across RGC types: some pairs of cell types exhibit totally non-overlapping response repertoires, and only a few exhibit substantial overlap. We further show that the diverse naturalistic signaling patterns can be used to distinguish the many RGC types, including cell types not as easily distinguished using artificial visual stimuli.

## Methods

### Tissue preparation

Retinas were obtained from macaque monkeys following terminal procedures conducted in compliance with Stanford Institutional Animal Care and Use Committee guidelines. Following enucleation, the eyes were hemisected and the vitreous was removed. Small retinal segments (approximately 2 mm × 3 mm) with the retinal pigment epithelium attached were dissected from regions 8–16 mm from the fovea, after which the choroid was trimmed to optimize tissue oxygenation. Retinal eccentricity was measured with a precision of 1–2 mm. Distance and the angle from the fovea were converted into horizontal and vertical visual degrees as described in Chichilnisky and Kalmar (2002). For the six out of seven recordings used in this work, the locations were recorded and corresponded to 36°–54° of eccentricity (temporal equivalent). In particular, horizontal and vertical eccentricity was: (1) 40°, 0°; (2) −14°, 53°; (3) −31°, 54°; (4) −69°, −40°; (5) 40°, 0°; and (6) 0°, 60°. Positive numbers correspond to temporal or superior locations, negative numbers correspond to nasal or inferior locations.

### Multi-electrode array recordings and spike sorting

Retinal ganglion cell activity was recorded using custom multi-electrode arrays (MEAs) with 512 electrodes arranged in a 16 × 32 isosceles triangular grid with a 60 μm separation between rows and between electrodes in a row, covering roughly 1 mm × 2 mm. The retina was mounted RGC side down onto the MEA and secured with a permeable membrane. During recordings, the tissue was continuously perfused with oxygenated Ames’ solution (Sigma, St. Louis, MO, USA) maintained at 31°C–33°C. Voltage signals were band-pass filtered, amplified, and digitized at 20 kHz using custom electronics (Litke et al., 2004). Spike sorting was performed with Kilosort2 (Pachitariu et al., 2024), and only cells meeting rigorous quality criteria (no refractory period violations, distinct electrical images, and consistent receptive field properties across cells of each identified type) were included in the analysis. Because some identified cell types exhibited incomplete receptive field mosaics due to unrecorded cells or spike sorting limitations, the number of identified cells per type does not reflect their true density. In the retinal periphery, cell types other than parasol, midget, or small bistratified each constitute 1.1%–4% (Kim et al., 2022) of the entire RGC population.

### Visual stimulation

Two types of visual stimuli were presented on a computer display and focused onto the photoreceptor layer. The display intensity produced on average 800–2,200, 800–2,200, and 400–900 photoisomerizations per second for the L, M, and S cones respectively. For cell classification and receptive field mapping, a series of 4–8 white noise stimuli (flickering checkerboards) was presented, each lasting 30–60 min and with differing pixel size and refresh time. In these stimuli, the contrast of each pixel was selected randomly and independently over space and from a binary distribution at each refresh. In some cases the three display primaries were modulated independently, in other cases the three primaries were yoked. For naturalistic stimulation, grayscale images from the ImageNet dataset (Fei-Fei et al., 2010) were used. Each image was presented for 100 ms, followed by 400 ms of a uniform gray background at mean luminance, a design intended to isolate individual responses and minimize adaptation. A total of 10,000 unique images was shown. A block of 150 repeated images was presented after every 1,000 unique images to monitor recording stability.

### White noise analysis

The responses to white noise were analyzed as described elsewhere (Field et al., 2007; Rhoades et al., 2019; Kling et al., 2024). Briefly, the spike-triggered average (STA) from the white noise stimulus was used to extract spatial and temporal response properties, facilitating cell type classification based on time course, inter-spike interval distributions, and mosaic (Kling et al., 2024). Cells were then categorized into known types (e.g., ON/OFF parasol, midget, smooth monostratified types, and broad thorny type) (Kling et al., 2024) and other putative types based on similarity of these parameters and the mosaic organization of receptive fields.

To compute the time courses, significant pixels in the STA were identified as follows. First, the dominant STA frame was determined by selecting the frame with the maximal absolute pixel intensity. Within that frame, pixels were considered significant if their intensity exceeded four times the robust standard deviation calculated across all pixels. These significant pixels were then grouped by polarity (sign), and their spatial average was computed for each STA frame separately for each group, resulting in two time courses for each display primary (only the dominant polarity is shown). For the blue and green channels, the time course corresponding to the dominant polarity (i.e., with the highest absolute amplitude) was selected.

**FIGURE 1:**
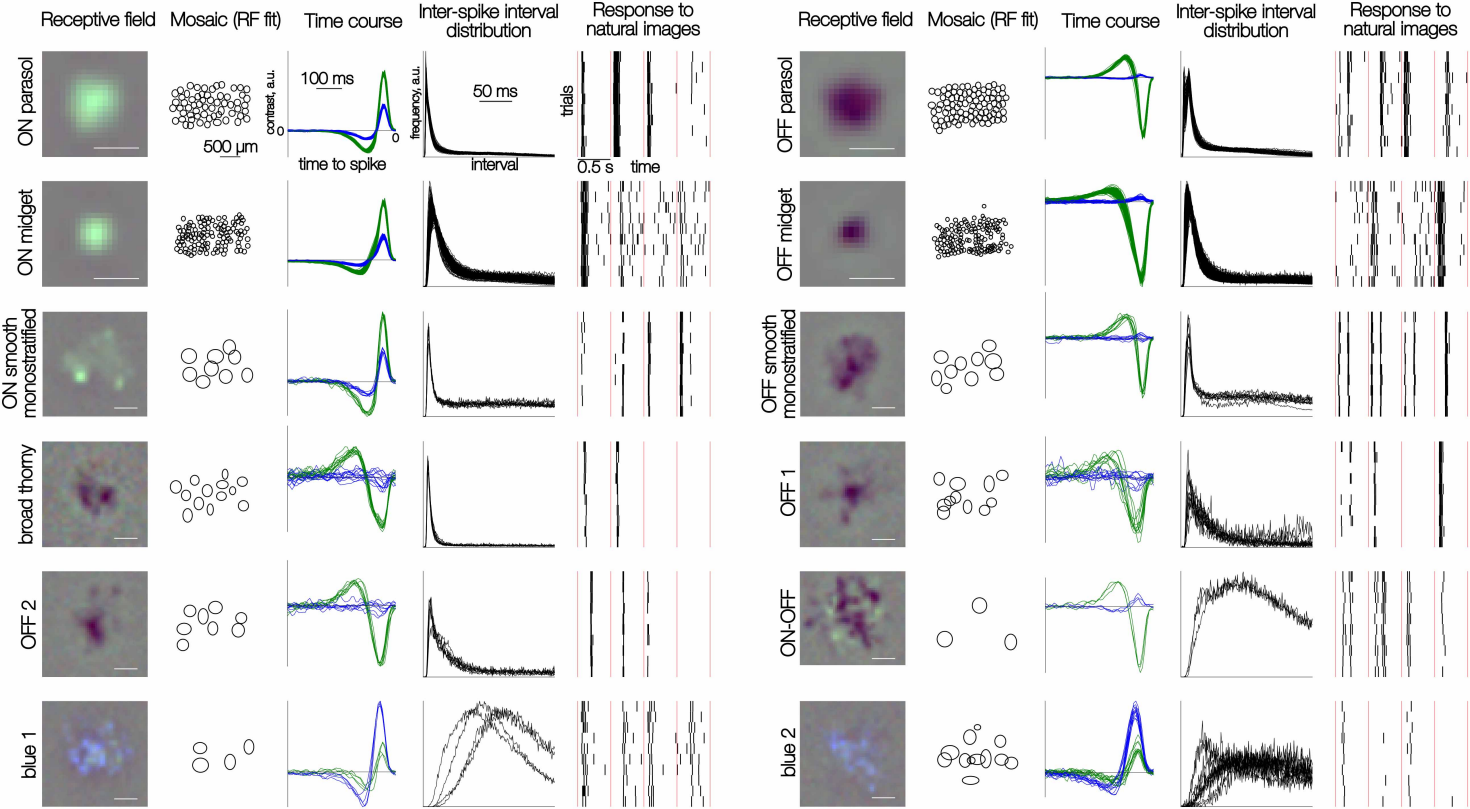
Distinct responses to white noise and natural images in 12 RGC types. Each row of five panels shows results for multiple cells of one type. **(Left to right)** The spatial RF of one cell obtained from the spike-triggered average (see section “Methods”); the mosaic of elliptical fits to the RF profile; time courses for green and blue display primaries obtained from the spike-triggered average; inter-spike interval distributions in the presence of the white noise stimulus; rasters of spike responses to 10 repeated trials of 4 consecutive natural images. Each image was presented for 100 ms; red vertical lines separate distinct image trials (0.5 s trial duration). Scale bar for RFs: 200 μm, for mosaics: 500 μm.

An elliptical fit of the spatial receptive field was obtained by fitting a 2D Gaussian to the STA frame with the maximum signal amplitude. The 2-standard deviation contour of this fit is shown in Figure 1 to illustrate the mosaic organization.

### Natural image analysis

**FIGURE 2:**
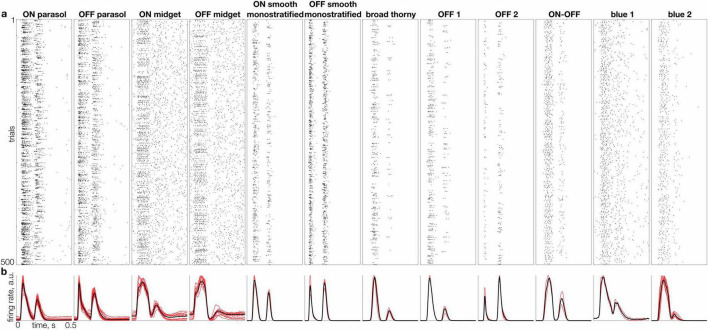
Individual and average responses of 12 RGC types to natural images. **(a)** Raster of responses to 500 randomly selected flashed images (rows), for a representative cell of each distinct type (columns). **(b)** Mean firing rate across the full set of 10,000 images (L2-normalized) for multiple cells of each type (red traces) and average for all cells of each type (black trace).

For naturalistic stimuli, spike responses were aligned to stimulus onset, smoothed with a Gaussian kernel (σ = 10 ms) and averaged over a 500 ms window to obtain the evoked mean firing rate for each cell (Figure 2). To capture the full repertoire of responses, two temporal features for each response were computed: latency (defined as the time to peak amplitude) and duration (defined as the time from the peak until the response decayed to 25% of its maximum). These features were calculated for a random subset of 1,500 responses per cell, after excluding trials with zero or one spikes, to ensure only stimulus-driven responses were analyzed. The results were robust to resampling and to varying the subset of selected trials between 500 and 3,000 images (across cells, the total number of trials after filtering for the number of spikes varied between 2,000 and 9,500). Alternate definitions of latency (the time at which half the spikes in the trial were recorded) and duration (full width at half-height for the largest peak) yielded results similar to those obtained with the original definitions. The difference between metrics after normalization was similar in magnitude to the difference across resamplings of 1,500 trials.

The distance between the response repertoires of two cells was quantified by (*latency*^2^ + *duration*^2^)^1/2^. For each response from cell 1, the *k* smallest distances (typically *k* = 10) to responses from cell 2 were identified, and the inverse of their average was taken as a similarity score.

### Two-dimensional representation of cell properties

**FIGURE 3:**
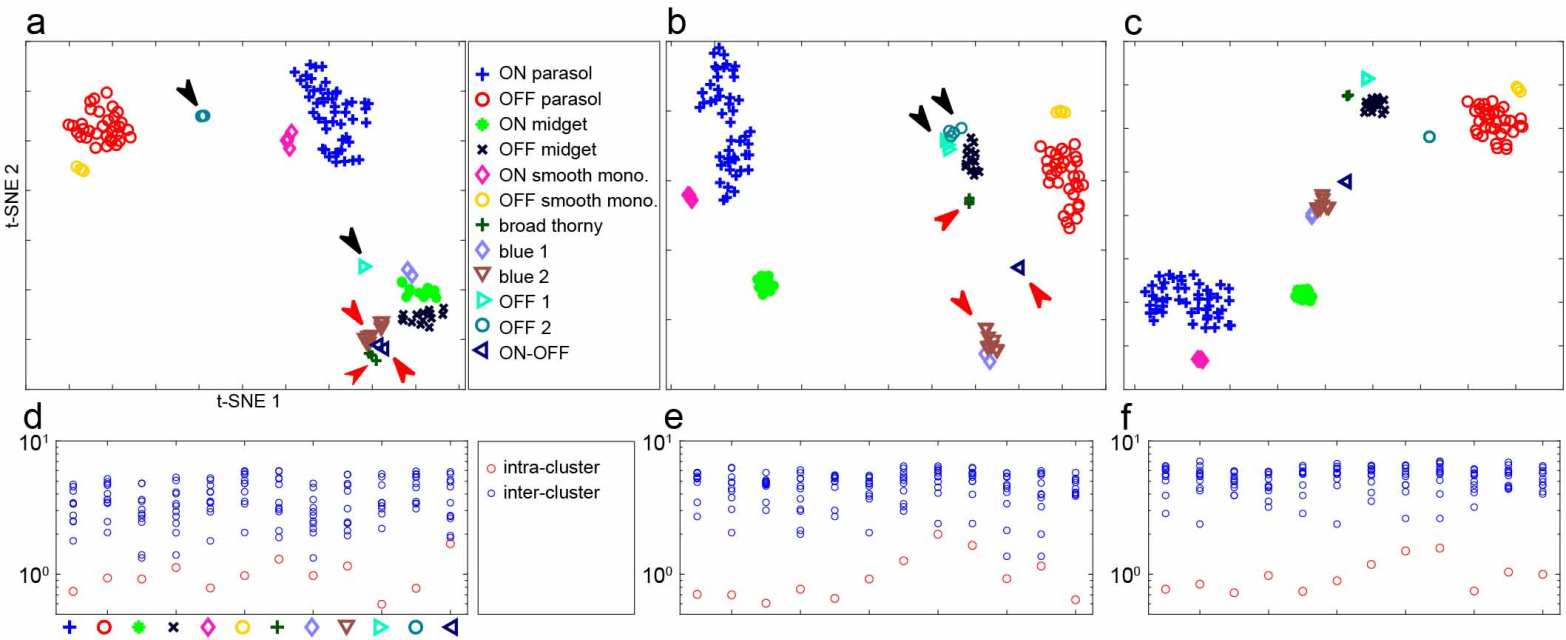
Separability of RGC light responses in one recording. **(a)** t-SNE representation of recorded firing rates (see Figure 2) of 141 RGCs in response to flashed natural images. Cell types were identified using responses to white noise stimuli (Figure 1, see section “Methods”). Black arrows point to well-separated clusters, red arrows point to clusters that are more difficult to distinguish. The axes of t-SNE plots are not readily interpretable; thus, this representation of clustering serves primarily as an illustration. **(b)** t-SNE representation of spike-triggered average time course and inter-spike interval (see section “Methods”) of the same cells with white noise stimuli. Black and red arrows point to the same groups of cells as in panel **(a)**. **(c)** t-SNE for the same cells based on both natural image and white noise data. **(d)** Mean intra-cluster and inter-cluster Euclidean distances of the first 10 principal components of responses to natural images, normalized to the mean intra-cluster distances (see section “Methods”). **(e)** Same, for spike-triggered average time course and interspike interval with white noise stimuli. **(f)** Same, for combined natural image and white noise data.

To visualize cell type clusters (Figures 3a–c), principal components analysis was applied to several groups of parameters: (1) normalized time course of the blue and green display primaries (Figure 1, column 3) concatenated with the normalized interspike interval distribution obtained during white noise stimulation (Figure 1, column 4): total vector length 160 values per cell; (2) normalized average response time courses (Figure 2b) for natural image stimuli (see above): total vector length 500 values per cell; and (3) a concatenation of 1 and 2, total vector length 660 values per cell. All normalizations were performed with the L-2 norm. The t-distributed Stochastic Neighbor Embedding (t-SNE; van der Maaten and Hinton, 2008) was used on the first 10 principal components to reduce the data into two dimensions.

To estimate cluster separability (Figure 3d–f), pairwise Euclidean distance was computed using the first 10 principal component scores of response features as described above, across all cells selected for the analysis in a recording, separately for each condition (natural image only, white noise only, and combined). For each condition, intra-cluster distance was defined as the mean pairwise distance between all cells of the same type. Inter-cluster distance was defined as the mean pairwise distance between all cells of one type and all cells of a different type. All distances were then normalized by the mean of intra-cluster distances within the corresponding condition.

## Results

### Kinetics of mean response to natural images vary substantially across cells

The visually evoked spiking activity of hundreds of peripheral primate RGCs was recorded *ex vivo* on a custom 512-electrode recording system (Litke et al., 2004; Frechette et al., 2005). To characterize light response properties and classify cells, a white noise stimulus (flickering checkerboard) was used (see Chichilnisky, 2001; Field et al., 2007; Kling et al., 2024; Rhoades et al., 2019; Litke et al., 2004). Cell types were distinguished based on the STA stimulus time courses and the interspike interval distribution observed during white noise stimulation. The accuracy of cell type classification was confirmed by the mosaic organization of the receptive fields of cells of each type (Kling et al., 2024; Figure 1). Note that the number of recorded cells of each type does not necessarily reflect their true density in the retina because of sampling biases associated with extracellular recordings (see section “Methods”).

To explore the behavior of the diverse RGC types in naturalistic conditions, the responses to 10,000 flashed grayscale natural images were recorded. Each image was presented for 100 ms, followed by 400 ms of a gray background with intensity equal to the mean intensity of all images, a protocol designed to temporally separate responses to distinct stimuli and to minimize adaptation. A specific sequence of 150 images was repeated after each block of 1,000 unique images to monitor response stability. All cells selected for subsequent analysis exhibited a reproducible pattern of spiking response to the repeated images (Figure 1). As expected, the responses of a given cell to distinct images were, in general, markedly different (Figure 1). Also, as expected, a given image elicited clearly distinct response kinetics in different cells.

To capture the typical kinetics of responses to natural images produced by each cell, its measured responses to all images were aligned to the stimulus onset (Figure 2a), temporally smoothed, and averaged across images (Figure 2b). Despite the variability of responses across images (Figure 1), the mean response exhibited distinct average kinetics in each cell type (Figure 2b, black lines) and similar kinetics in cells of the same type (Figure 2b, red lines). This consistency indicates that the average response to a large set of natural images reveals a response signature representative of each cell type, in a similar manner as the STA signature obtained with white noise stimuli (Kling et al., 2024).

**FIGURE 4:**
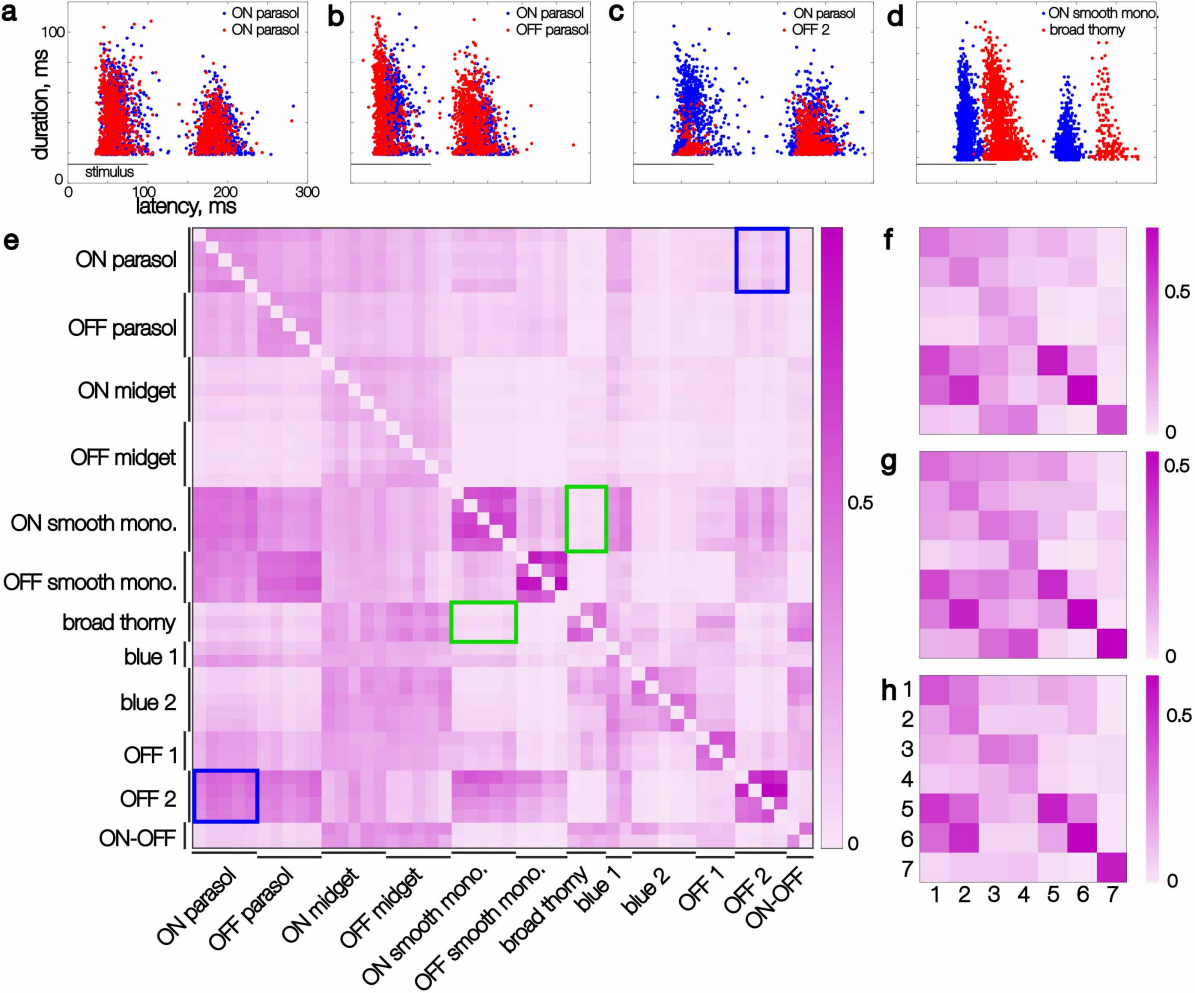
Kinetic features extracted from recorded responses of pairs of simultaneously recorded cells and their similarity. **(a–d)** Each point represents the latency and duration of the response of one cell to a single flashed image, smoothed in time (see section “Results”). Image presentation period is marked with a horizontal line. **(a)** Two ON parasol cells (red and blue dots, respectively). **(b)** An ON parasol and an OFF parasol cell. **(c)** An ON parasol and an OFF 2 cell. **(d)** An ON smooth monostratified and a broad thorny cell. **(e)** Similarity scores (see section “Results”) for individual cells of 12 distinct types in a single recording, sorted by type. At most five cells per type are shown. The rows show the similarity score with the cell listed on the ordinate as the reference cell. Blue rectangles highlight the asymmetrical similarity of ON parasol to OFF 2 cells compared to the reverse. Green rectangles highlight symmetric low scores between broad thorny and ON smooth monostratified cells. **(f)** Similarity scores for the same recording as panel **(e)**, averaged across each cell type, for 7 identified types (1 through 7: ON parasol, OFF parasol, ON midget, OFF midget, ON smooth monostratified, OFF smooth monostratified, and broad thorny). **(g,h)** Same as panel **(f)**, for two additional datasets.

The average kinetics of responses to natural images exhibited several trends across cell types. First, as expected, parasol cell responses were more transient than midget cell responses. Second, for morphologically paired cell types (e.g., ON vs. OFF parasol cells), OFF cells tended to have a shorter time to peak on average than ON cells (see Figure 4a), in agreement with some previous work (Gollisch and Meister, 2008) (but see Chichilnisky and Kalmar, 2002). Third, low-density cell types exhibited a degree of response transience more similar to parasol than to midget cells. Interestingly, most of the low-density cell types – ON and OFF smooth monostratified, broad thorny, OFF 1 and OFF 2 types – exhibited more transient responses than parasol cells, while no cell types with more sustained responses than midget cells were identified. Fourth, some cell types – such as parasol and smooth monostratified cells – exhibited strong responses to both image onset and offset (corresponding to the two peaks in Figure 2b), while other cell types – including OFF midget and blue 2 cells – responded predominantly to image onset or offset. These observations reveal the diversity of the average natural visual signal across primate RGC types.

### Cell types can be identified by the average kinetics of responses to natural images

To test if the average response signature could be used to reliably classify distinct RGC types, t-SNE (van der Maaten and Hinton, 2008) was applied to the first 10 principal components of the average response profiles of all recorded cells. The results revealed that cells largely clustered into well-defined groups (Figure 3b) corresponding to the types determined above (Figure 1). For a direct comparison, t-SNE applied to the first 10 principal components of the STA time course concatenated with the interspike interval distribution obtained using white noise stimulation also produced a set of clusters separating the identified cell types, as expected (Figure 3a, see section “Methods”).

Interestingly, however, the two distinct types of stimuli more clearly and reliably distinguished different sets of RGC types. For example, as viewed in the t-SNE representation, white noise data did not reliably discriminate between cells of two OFF-dominated types that had similar inter-spike interval distributions and STA time courses (Figure 3b, black arrows; see Figure 1, OFF 1 and OFF 2 types) – these cells were separated into two types in the preceding analysis (Figure 1) largely because they formed two overlapping mosaics (Kling et al., 2024). However, with naturalistic stimuli, these two cell types were readily distinguished (Figure 3a, black arrows). Conversely, two cell types that produced similar mean responses to natural images (Figure 3a, red arrows) were readily distinguished by white noise stimulation (Figure 3b, red arrows). A combined t-SNE analysis, applied to concatenated data from the two types of visual stimulation, yielded a clearer separation of cell types (Figure 3c). This result suggests that natural image responses provide discriminative power that augments the discriminative power provided by white noise stimuli. Note, however, that the t-SNE representation can be difficult to interpret and may not fully capture the quantitative separability of cell types.

To quantitatively test the separability of cell types obtained using both types of stimuli, intra-cluster distances were compared to inter-cluster distances in the original principal components representation without the t-SNE dimension reduction (Figures 3d–f). Each stimulus type alone revealed that for the most part the inter- and intra-cluster distances were non-overlapping (Figures 3d, e), but combining the data from both stimulus types yielded a more clear separation (Figure 3f). Using half of the white noise data and half of the natural scenes data for the combined analysis had no discernible effect on the results (not shown), confirming that the enhanced separability was not attributable to using more data.

### Repertoire of responses to natural images is cell-type specific

Although the average response kinetics provide a useful summary, they do not reveal the full repertoire of responses produced by each cell to many images. For example, a similar average response in two cells or cell types could be achieved with very different individual responses. Therefore, to understand the similarity of natural image signaling between cells and cell types, a simple comparison was performed on the repertoire of responses produced by pairs of cells.

Specifically, the latency and duration of each response was computed, and the collection of latency and duration values across images was compared directly for pairs of cells (see section “Methods”). A range of relationships between cell types was observed: highly overlapping response repertoires (Figure 4a), partially overlapping repertoires (Figure 4b), subset repertoires (Figure 4c), and fully distinct repertoires (Figure 4d). Repertoires of cells of the same type typically fully overlapped with each other. The repertoires of cells of paired ON and OFF types (e.g., ON and OFF parasol) tended to overlap slightly less, due to the shorter latencies in OFF types (Figure 4b). Interestingly, some cell type pairs revealed complex relationships between their response repertoires. For instance, the onset responses of an OFF-dominated cell (OFF 2) formed a subset of the onset responses of an ON parasol cell, especially in terms of duration, while the collections of offset responses were more similar (Figure 4c). Finally, some cell types, such as ON smooth monostratified and broad thorny cells, appeared to have a fully distinct set of latencies – even the slowest responses of a ON smooth monostratified cell reached peak amplitude earlier than the fastest responses of a broad thorny cell. These findings reveal a complex retinal code during naturalistic stimulation.

To quantify similarity in response latency and duration across cell pairs, the inverse of the mean Euclidean distance to the most similar responses was computed (see section “Methods”). This measure is asymmetric: if the responses of cell A form a subset of the responses of cell B (e.g., Figure 4c), the similarity of A to B will be high, while the similarity of B to A will be lower (e.g. compare lower and upper triangles of Figure 4e).

The response repertoire similarity was consistent for all cell pairs composed of specific types within a recording, as revealed in the block-wise structure of the similarity matrix (Figure 4e). For instance, each broad thorny cell exhibited low similarity to each ON smooth monostratified cell, and vice versa (Figure 4e, green frames). On the other hand, each pair of OFF 2 and ON parasol cells exhibited consistently asymmetrical scores (Figure 4e, blue frames). The similarity score also revealed internal variability of the response repertoire: cells with a tighter distribution of response parameters exhibited a higher within-type similarity. For example, within-type pairs of ON and OFF smooth monostratified and broad thorny cells had higher similarity than within-type pairs of parasol, midget, and blue 1 and blue 2 cells. The type-specific response repertoire was also robust to variability between animals and experiments, as revealed in the consistent pattern of the per-type similarity matrix across experiments for cells of known types (ON and OFF parasol, midget, smooth monostratified, and broad thorny) (Figures 4f–h). Thus, the diversity of *average* response properties of cells of different types also applies to the entire *repertoire* of their responses, within and across experimental preparations.

## Discussion

Large-scale recordings from diverse types of macaque RGCs showed that both the mean response and the full repertoire of responses to natural images are highly stereotyped within each cell type and often distinctive in different cell types, including the less-understood low-density cell types. A few cell types exhibited very similar response repertoires and averages to one another, but most types differed substantially from others, in a consistent manner across retinas. Overall, each RGC type exhibited an intrinsic functional signature that is maintained under the complex conditions of naturalistic stimulation.

The distinctions observed have practical implications for understanding cell type diversity in the retina, because responses to natural images highlighted distinctions between types (Figure 3a) that were not as readily visible with white noise stimulation (Figure 3b), a standard method for cell type identification in large-scale recordings (Field et al., 2007; Kling et al., 2024; Rhoades et al., 2019). A possible reason for this is that naturalistic stimulation engages non-linear processing mechanisms (Freedland and Rieke, 2022; Turner and Rieke, 2016; Karamanlis et al., 2023) that generate richer temporal patterns of response. However, the practical utility of this finding may be limited because responses to natural images often present challenges for spike-sorting due to highly correlated responses across cells. Synthetic visual stimuli (e.g., Freedland and Rieke, 2022) could potentially leverage some of the advantages of natural images without compromising spike sorting as much, an area for further exploration.

The stimuli used in this study were presented at a low photopic light level for both white noise and natural image conditions. In the future experiments, testing different photopic and scotopic light levels could provide insight into differential light adaptation across cell types and perhaps further enhance their separability.

The striking distinctions in response repertoires observed during natural stimulation suggest the possibility that downstream mechanisms in the brain could exploit these patterns during development for cell type specific refinement of synaptic contacts. Many studies of visual system development focus on the molecular mechanisms that contribute to specific connectivity in retinal targets (Sperry, 1963; Huberman et al., 2008; Feldheim and O’Leary, 2010). However, a large body of work also points to the importance of visually driven activity in segregating retinal inputs to central structures (Sengpiel and Kind, 2002; Huberman, 2007). Although a dominant theory is that correlated firing over space is a driving factor (Wong, 1999; Torborg and Feller, 2005; Xu et al., 2016), the distinctive natural patterns of activity in different RGC types could also play a role. The present results show that there is enough distinction in response repertoires to support such a mechanism.

Some of the distinctions between cell types were consistent with what would be predicted based on previous studies using simpler non-naturalistic stimuli such as white noise, gratings, and contrast steps (Chichilnisky, 2001; Turner and Rieke, 2016; Soto et al., 2020; Karamanlis et al., 2023). Specifically, midget RGCs displayed more sustained responses than parasol RGCs (Soto et al., 2020; Chichilnisky and Kalmar, 2002), ON and OFF RGCs of morphologically matched types (midget, parasol, and smooth monostratified) had very similar response repertoires and mean responses, and OFF RGCs tended to have slightly shorter response latencies than ON RGCs of morphologically matched types (Gollisch and Meister, 2008). Thus, some of the major cell type distinctions seen in earlier studies are applicable to natural vision.

However, some distinctions between cell types, particularly the less-studied types, have not been reported previously, and could potentially be important for understanding their role in natural visual signaling. The response latencies of smooth cells varied little across images compared to the latencies of other cell types (Figure 4), including parasol cells, an invariance that could support a role in signaling the timing of events in the visual scene to the brain. Some less-studied RGC types exhibited consistent response durations across different images, unlike midget and parasol cells which had response durations that varied strongly with image content (Figure 4), and several cell types exhibited more transient responses than parasol cells (Figure 2). The mechanisms for these distinctions in response kinetics across cell types are not known. In principle, such differences could arise from different spike generation mechanisms (Wienbar and Schwartz, 2022), synaptic input properties (Awatramani and Slaughter, 2000), or strong inhibitory input from amacrine cells (e.g., Puller et al., 2015; Bordt et al., 2021). However, the specific visual features that drive responses in different cell types and the underlying mechanisms will require further investigation.

Three primary limitations of the present work could be important for its interpretation. First, the stimulus set consisted of grayscale flashed natural images, which may not fully engage the dynamic response properties of certain RGC types. Second, the use of only two kinetic features (latency and duration) to analyze cell type distinctions may not reveal the full diversity of responses. Future studies incorporating stimuli with color, object motion, optic flow, as well as analysis of a broader range of response features such as burstiness and ON/OFF asymmetry will be important to understand the full range of RGC responses across cell types in natural viewing conditions. Finally, the present analysis was limited to the mid-peripheral retina because of the technical challenges of large-scale recording near the fovea. Comparison to the central retina would provide valuable insights into the relevance of the present findings for high-acuity vision.

## Data Availability

The raw data supporting the conclusions of this article will be made available by the corresponding author, upon reasonable request.
